# Usefulness of maximum intensity projection images of non-enhanced CT for detection of hyperdense middle cerebral artery sign in acute thromboembolic ischemic stroke

**DOI:** 10.1007/s11604-022-01289-8

**Published:** 2022-05-25

**Authors:** Sota Oguro, Shunji Mugikura, Hideki Ota, Seiji Bito, Yuta Asami, Wataru Sotome, Yoshiaki Ito, Hideki Kaneko, Kazuyo Suzuki, Nobuya Higuchi, Kei Takase

**Affiliations:** 1 Department of Diagnostic Radiology, Tohoku University Hospital, 1-1 Seiryo-machi, Aoba-ku, Sendai, Miyagi 980-8574 Japan; 2 Department of Internal Medicine, Tokyo Medical Center, Tokyo, Japan; 3 Department of Diagnostic Radiology, Tokyo Medical Center, Tokyo, Japan

**Keywords:** Maximum intensity projection, Stroke, Hyperdense middle cerebral artery sign, Computed tomography

## Abstract

**Purpose:**

To compare the sensitivity of the hyperdense middle cerebral artery (MCA) sign between maximum intensity projection (MIP) and conventional averaged images in patients with acute focal neurological deficits with acute thromboembolic MCA occlusion (MCA occlusion group) and patients with acute focal neurological deficits without MCA occlusion (control group).

**Materials and methods:**

Initial computed tomography (CT) scans on admission were reconstructed with 5 mm thickness at every 3 mm interval for averaged and MIP images from 1 mm thickness non-contrast axial source images. Images were obtained from 30 cases each in the MCA occlusion and control groups. The CT values in the region of interests (ROIs) on the affected and unaffected sides of the MCA were compared. To compare CT values among subjects, the CT values were normalized by obtaining a ratio on the affected and unaffected sides, and the normalized CT values were analyzed using the receiver operating characteristic (ROC) curve.

**Results:**

The hyperdense MCA sign was visually detected on MIP images in 90% cases and on 5 mm averaged images in only 57% cases in the MCA occlusion group. Based on the ROC analysis of the normalized ratio on the affected and unaffected sides, area under the curve of MIP image and averaged image was 0.941 and 0.655, respectively. On MIP images, the optimal threshold of the ratio on the affected and unaffected sides was 1.152 (sensitivity: 90.0%, and specificity: 93.3%).

**Conclusion:**

The hyperdense MCA sign sensitivity on 5 mm MIP images was significantly higher than that on conventional 5 mm averaged CT images. This could be useful for the early initiation of proper therapy for patients with acute focal neurological deficits.

## Introduction

Non-enhanced computed tomography (CT) scans are widely used for the diagnosis of neurological deficits caused by acute stroke. When there is no brain hemorrhage, a hyperdense region in the horizontal portion of the middle cerebral artery (MCA) on the ipsilateral side (hyperdense MCA sign) suggests acute occlusion. A hyperdense MCA sign is caused by the presence of an intravascular thromboembolus, and the density on the ipsilateral MCA is higher than that on the contralateral MCA [1, 2]. This sign becomes visible earlier than the other early CT signs that includes parenchymal changes in the lentiform nucleus or insular ribbon [3]. Given that mechanical thrombectomy (MT) has become a standard therapy in acute stroke patients with the internal carotid artery or MCA occlusion, there is a growing demand for rapid access to the imaging examination that can confirm the presence of thromboembolic event. Several studies addressed the relationship between the presence of hyperdense MCA sign and prognosis after MT [4–6]. However, the use of the hyperdense MCA sign is limited because of the low sensitivity for the detection of thromboembolism [7]. Brain CT is commonly reconstructed at 4- or 5-mm thickness to obtain a good compromise between image noise and slice thickness, but it may be too thick to delineate a small high-density thrombus in the MCA because of a partial volume effect [8, 9]. Furthermore, a high hematocrit value under hemoconcentrated conditions increases the density in the blood vessel, which may lead to false-positive findings of hyperdense signs [10, 11]. Many attempts have been made to improve the sensitivity of the hyperdense MCA sign [8, 9, 12, 13]. Previous reports showed that an acute thromboembolus could be detected with higher sensitivity on 1 or 1.5 mm thin-section non-enhanced CT than on 5 mm non-enhanced CT [8, 9, 13, 14]. However, the increased number of images and the low signal-to-noise ratio have been reported as drawbacks of thin-section images [9]. In addition, visual comparison between bilateral MCAs on thin-section images is sometimes difficult and time-consuming because MCAs in a few diameters are not always demonstrated on the same planes, even when they are symmetrical. Therefore, a reliable method for quick evaluation of non-enhanced CT is desired in the emergency situations that requires MT.

Maximum intensity projection (MIP) images have been reported to be diagnostically useful over thin-section images when distinguishing hyperdense structures, such as vessels, nodules, and calcifications, with respect to surrounding tissues [13]. Signal-to-noise ratio of MIP images should be higher than that of thin-section images [15]. Thus, we considered the use of MIP images alone to improve the sensitivity of small thromboembolus in emergency situations. To the best of our knowledge, few studies have been reported on MIP images [16].

The purpose of this retrospective study was to compare the sensitivity of the hyperdense MCA sign between 5 mm MIP images and conventional 5 mm averaged images in 30 patients with acute thromboembolic MCA occlusion and 30 control patients without MCA occlusion. As a quantitative analysis, CT values at MCA artery was also evaluated.

## Materials and methods

This retrospective study was approved by the Institutional Review Board. The requirement for informed patient consent was waived because of the retrospective nature of the study.

### Subjects

The patients with ischemic stroke were retrieved from February 1^st^ 2018 to June 30^th^ 2019 in a single-center. The patients were searched on the hospital reporting system (F-Report, Fujifilm Medical Systems, Tokyo, Japan). Inclusion criteria were as follows: (1) patients who presented with acute focal neurological deficits, such as hemiplegia and aphasia. (2) Patients who underwent non-enhanced CT and magnetic resonance imaging (MRI) within 7 days after onset. This study included 30 patients with acute thromboembolic MCA occlusion (MCA occlusion group) and 30 patients without MCA occlusion (control group).The number of patients was decided as 30 consecutive cases, and the cases of each group were collected starting from February 1st 2018 until 30 cases were found. In our hospital, non-contrast CT is performed first in patients with neurological deficit symptoms, followed by MRI and magnetic resonance angiography (MRA). The level of urgency of the MRI is determined by the attending physician based on the severity of symptoms.

Patients in the MCA occlusion group included 17 men and 13 women with a mean age of 85.0 ± 13.7 years (range 31–92 years), and those in the control group included 17 men and 13 women with a mean age of 79.5 ± 13.0 years (range 48–97 years) (± stands for standard deviation). Demographics and further characteristics, including stroke risk factors (smoking, hypertension, diabetes, ischemic heart disease, atrial fibrillation, and previous stroke), time from onset to MRI and CT imaging, mechanism of cerebral infarction, location of embolism, and National Institutions of Health Stroke Scales sores (NIHSS) were recorded.

Details for the image interpretation are described below.

### CT image acquisition

All brain CT examinations were performed by an 80-row multidetector CT (Aquilion Prime, Canon Medical Systems, Otawara, Japan). The scan parameters were as follows: tube voltage, 120 kVp; tube current, 300 mA; helical pitch, 51 (pitch factor, 0.637); field of view, 240 mm; and matrix size, 512 × 512. All images were reconstructed with filtered back projection. Initial CT scans on admission were reconstructed in 5-mm thickness with 3-mm reconstruction interval for averaged and MIP images from 1 mm thickness non-contrast axial source images using the CT scanner console.

### MR image acquisition

All patients were imaged with a 1.5 T MR scanner (Ingenia, Philips, Best, the Netherlands). Non-enhanced time-of-flight (TOF) MRA, single-shot echo planar diffusion-weighted (DW), and fluid-attenuated inversion recovery (FLAIR) images were obtained. For TOF MRA, the scan parameters were as follows: echo time (TE), 3.6 ms; repetition time (TR), 24 ms; flip angle 18°; matrix size, 248 × 183; field of view, 160 × 160 mm; slice-thickness, 1 mm; and acquisition time, 6 min 12 s. The scan parameters for DW images were as follows: TE, 81 ms; TR, 4000 ms; field of view, 220 × 246 mm; matrix size, 140 × 188; number of the slice, 21; thickness, 6 mm and acquisition time, 86 s. The scan parameters for FLAIR images were as follows: TE, 110 ms; TR, 10,000 ms; TI, 2700 ms; field of view 220 × 220 mm; matrix size, 260 × 189; slice-thickness, 6 mm and acquisition time, 210 s.

### MR image interpretation

The presence of MCA occlusion was diagnosed using MRA, and FLAIR images were also used to support the MRA findings of the MCA occlusion [17]. Two radiologists with 20 and 18 years’ experience (S.O., H.K.) evaluated all MR images by consensus. The review was conducted blinded to CT and clinical information.

### CT image interpretation

All CT datasets with 5-mm averaged images and 5-mm MIP images were evaluated by the two radiologists independently (S.O., H.K.). Final decision was reached through consensus. To mimic the routine clinical settings, ipsilateral sides of focal neurological symptoms were provided to the reviewers. The other clinical information and MR findings were blinded. Image review of the MIP and averaged images were performed visually and quantitatively in separate review sessions.

**Fig. 1:**
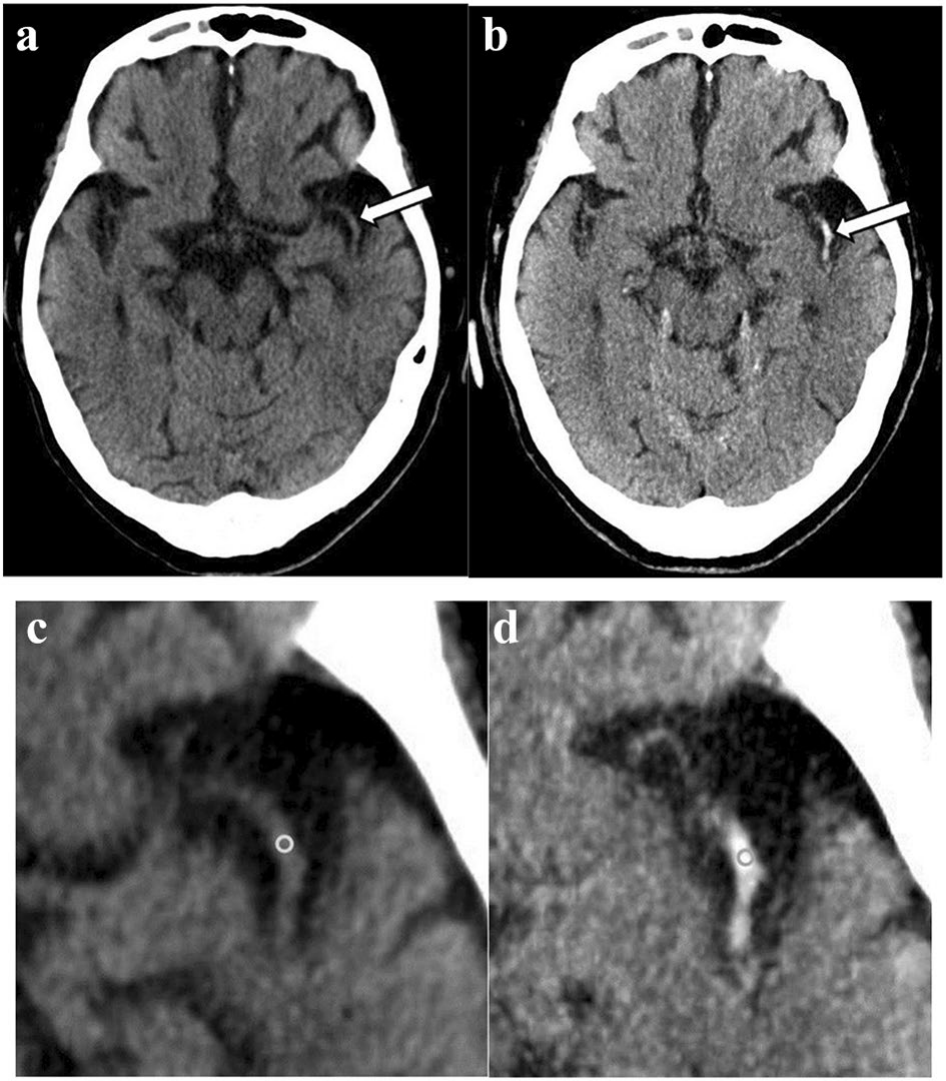
Case of hyperdense middle cerebral artery sign detected only on maximum intensity projection (MIP) images. On the 5-mm averaged image (**a**), the left middle cerebral artery did not show apparent hyper density (arrow). On the MIP image (**b**), the left middle cerebral artery appeared hyperdense (arrow). Window level (Hounsfield unit [HU]) and window width (HU) were preset as 30 HU/40 HU on 5 mm-thickness images and 40 HU/40 HU on MIP images, respectively. Region of interest (ROI) in the middle cerebral artery on cropped and enlarged images. The ROIs at the middle cerebral artery were 40 Hounsfield unit (HU) on 5-mm averaged image (**c**) and 75 HU on MIP image (**d**), respectively

A commercially available workstation (SYNAPSE Enterprise-PACS; Fujifilm Medical Systems, Tokyo, Japan) was used to evaluate CT images. Window level (Hounsfield unit [HU]) and window width (HU) were preset as 30 HU/40 HU on 5 mm-thickness images and 40 HU/40 HU on MIP images, respectively; these settings were adjusted if necessary. Hyperdense MCA sign was defined as a higher attenuation in the artery relative to the contralateral artery or to the adjacent parenchyma [7]. An example of MIP and averaged images of hyperdense MCA signs are shown in Fig. 1. For visual evaluation, the MIP and averaged images were reviewed with an interval of more than 1 month.

For quantitative evaluation, 2-mm-in-dimameter circular region of interests (ROIs) was placed on the ipsilateral and contralateral sides of the MCA, and mean HU values in the ROIs were recorded (Fig. 1). When the hyperdense MCA sign was not detected only on the averaged images, the ipsilateral ROI was placed in the same position as the MIP images. If the hyperdense MCA sign was not detected both on the averaged and MIP images in the MCA occlusion group, the location of the ROI was decided referring to the MRA occlusion site.

### Statistical analysis

Descriptive statistics are presented as means with standard deviation for normally distributed variables, medians with interquartile ranges for non-normally distributed variables, and numbers of cases (and percentages) per group for categorical variables. The inter-reader agreement of qualitative evaluation among reviewers was analyzed with Cohen’s κ: κ values of 0.01–0.20 indicated slight agreement; 0.21–0.40, fair agreement; 0.41–0.60, moderate agreement; 0.61–0.80, good agreement; and 0.81–1.00, excellent agreement. Patients’ demographics between the two groups were assessed using Fisher’s exact tests and Wilcoxon signed-rank tests. Sensitivity and specificity with Clopper-Pearson confidence intervals were calculated for detecting MCA occlusion by 5-mm averaged and MIP images. The diagnostic performance of both image datasets was statistically evaluated using the McNemar’s test. To compare CT values among subjects, the CT values were normalized by obtaining a ratio on the affected and unaffected sides, and the normalized CT values were analyzed using the receiver operating characteristic (ROC) curve. The analyses were performed using JMP software version 16.1.0 (SAS Institute Inc. Cary, NC, USA) and Microsoft Excel version 2019 (Microsoft Corporation, Redmond, WA, USA). The p-value of < 0.05 indicated statistical significance.

## Results

**Fig. 2:**
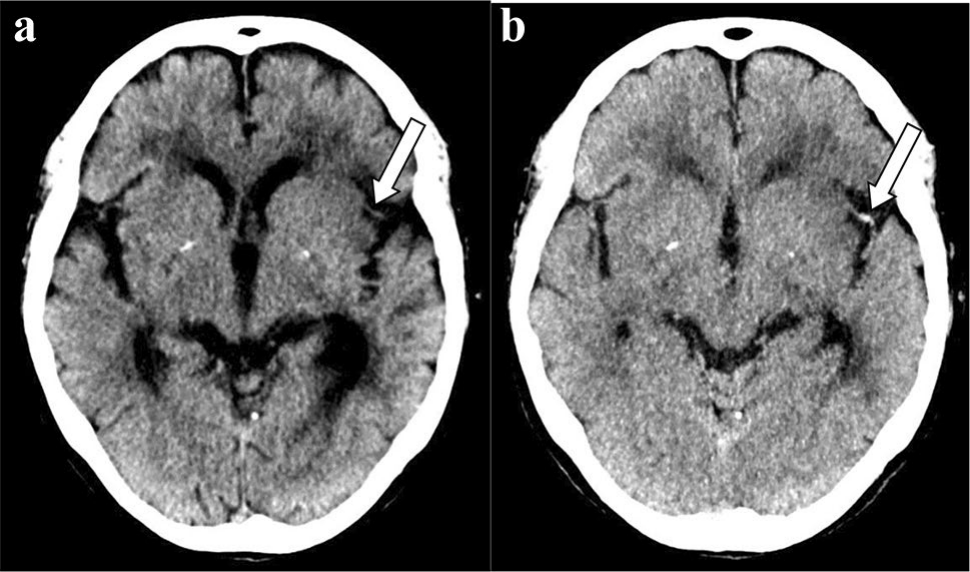
Another case of hyperdense middle cerebral artery sign detected only on maximum intensity projection (MIP) images. On the 5-mm averaged image (**a**), the left middle cerebral artery was surrounded by cerebrospinal fluid, and did not show apparent hyper density (arrow). On the MIP image (**b**), the left middle cerebral artery appeared hyperdense (arrow). Window level (Hounsfield unit [HU]) and window width (HU) were preset as 30 HU/40 HU on 5 mm-thickness images and 40 HU/40 HU on MIP images, respectively

Patients’ demographic backgrounds in each group are summarized in Table 1. Arterial fibrillation, time from onset to CT scan and MRI, and NIHSS were significantly different between the MCA occlusion and control groups. Patients included in the MCA occlusion group presented with acute focal neurological deficits, such as hemiplegia and aphasia, and had unilateral MCA occlusion on MRA performed on the same day. Acute infarction in the ipsilateral MCA was confirmed in all 30 cases in the MCA occlusion group, and the patients received intra-venous thrombolysis right after undergoing MRI and MRA. Patients in the control group also presented with focal neurological deficits, but without any MCA steno-occlusive lesions on MRA performed within 3 days from the onset. Twenty of the 30 cases showed acute small cortico-subcortical or lacunar infarction on DW images. The sensitivity and specificity with 95% confidence intervals for the detection of MCA occlusion were 90% (0.735, 0.979) and 100% (0.88, 1.00) for MIP images and 56.7% (0.384, 0.745) and 100% (0.88, 1.00) for averaged images, respectively. Review of MIP images demonstrated significantly higher sensitivity for the detection of MCA occlusion than review of averaged images (p < 0.01). The inter-reader agreement of qualitative evaluation among the reviewers was 1. No hyperdense MCA sign was recorded in the control group for either MIP or averaged images. The locations of the identified hyperdense MCA sign by CT matched with the MRA occlusion site in all cases. Parenchymal changes in the lentiform nucleus or insular ribbon were observed in five cases. Two typical cases in which the hyperdense artery sign was detected only on the MIP image are shown in Fig. 1 and 2.

**Table 1:** Patients’ background

	MCA^a^ occlusion group	Control group	
Total number	30	30	
Smoking	8	7	*p* = 1
Hypertension	22	18	*p* = 0.41
Diabetes mellitus	11	11	*p* = 1
Ischemic heart disease	8	3	*p* = 0.18
Arterial fibrillation	13	1	*p* < 0.01
Previous ischemic stroke	10	10	*p* = 1
Time from onset to CT^b^ scan (hour)	2.2 ± 1.5	8 ± 7.5	*p* < 0.01
Time from onset to MRI^c^ (hour)	3.5 ± 1.6	27.0 ± 23.5	*p* < 0.01
Location of embolism on magnetic resonance arteriography			
M1	25	0	
M2	5	0	
NIHSS^d^	16.7 ± 10.7	4 ± 3.5	

^a^MCA: Middle cerebral artery

^b^CT: Computed tomography

^c^MRI: Magnetic resonance imaging

^d^NIHSS: National Institutions of Health Stroke Scales

National Institutions of Health Stroke Scales was obtained from 20 cases in MCA occlusion group and 10 cases in control group

**Fig. 3:**
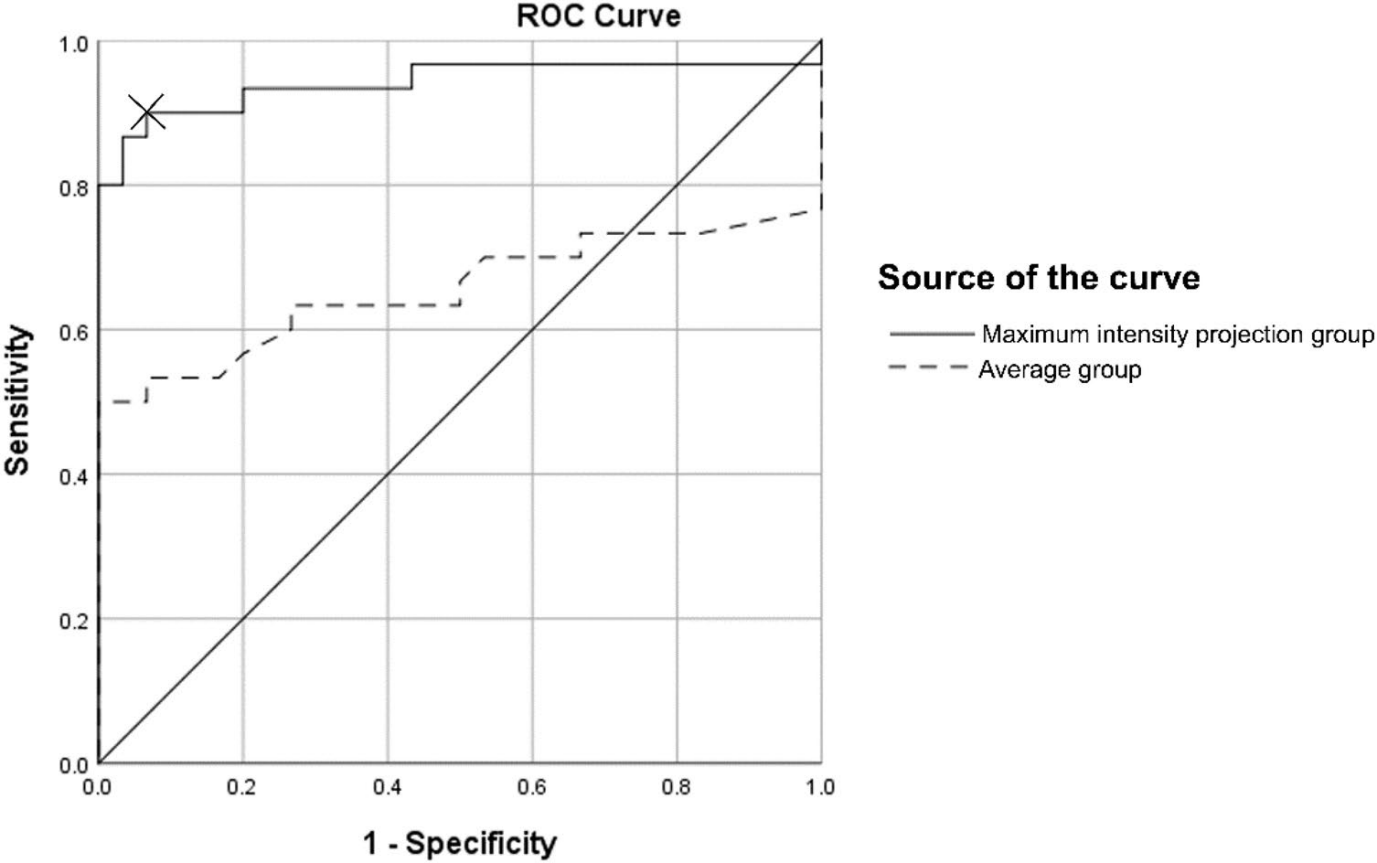
Receiver operating characteristics (ROC) analysis of the normalized ratio on the affected and unaffected sides. A cross mark (X) denotes the cut-off point (1.152) with the highest diagnostic performance

The ROIs of the MCA on the affected and unaffected sides in both the MCA occlusion and control groups are listed in Table 2. Based on the ROC analysis of the normalized ratio on the affected and unaffected sides, area under the curve (AUC) of MIP image and averaged image was 0.941 and 0.655, respectively (Fig. 3). On MIP images, the optimal threshold of the ratio on the affected and unaffected sides was 1.152 (sensitivity: 90.0%, and specificity: 93.3%).

**Table 2:** Computed tomography values of regions of interest measured in the middle cerebral artery (MCA) on averaged and maximum intensity projection images from the MCA occlusion and control groups

MCA occlusion group				Control group			
	Affected side*	Unaffected side*	Ratio*		Affected side*	Unaffected side*	Ratio*
Averaged	40.1 ± 8.4 HU	32.8 ± 3.7 HU	1.37 ± 0.19	Averaged	34.8 ± 4.6 HU	35.1 ± 3.5 HU	1.07 ± 0.06
MIP	61.9 ± 8.2 HU	44.8 ± 3.8 HU	1.23 ± 0.26	MIP	47.2 ± 4.7 HU	47.3 ± 4.3 HU	1.05 ± 0.05

*HU* Hounsfield unit, *MCA* middle cerebral artery, *MIP* maximum intensity projection

^*^Ratio: The CT values are normalized by obtaining a ratio on the affected and the unaffected sides on each patient

## Discussion

The results of this study indicate that the sensitivity of the hyperdense MCA sign on MIP images was significantly higher than that on averaged images. Hyperdense sign was first reported as “hyperdense MCA sign,” and the usefulness of the hyperdense sign have been reported especially in MCA [1, 6]. Furthermore, in terms of the frequency of acute occlusions, MCA occlusions are more common than anterior cerebral artery, posterior cerebral artery, and vertebral and basilar arterial occlusions [22], and endovascular thrombectomy is strongly recommended for acute cerebral large vessel occlusion of the internal carotid artery or M1 segment of the middle cerebral artery [23]. Therefore, we focused on only the hyperdense MCA sign. Gadda et al. showed that narrowing the window width could improve the sensitivity of the hyperdense MCA sign and discussed the usefulness of thin-section and MIP images [13]. Multiplanar images from thin-section CT have also been reported as useful. However, thin-slice images inhere low signal-to-noise ratio (SNR), and noise reduction techniques, such as model-based iterative reconstruction (MBIR), may contribute to increased sensitivity of the hyperdense MCA sign [9]. More recently, artificial intelligence studies of hyperdense MCA sign have been performed [12, 21]. However, there are important technical differences between CT equipment and between manufacturers, longer reconstruction time is needed for MBIR, longer calculation time is needed for artificial intelligence and these techniques or methods have not been widely used.

A small high-density area on the original 1 mm-thickness images is obscured on 5-mm averaged images due to partial volume effects. Whereas, the highest CT value in the high-density areas keeps contrast to the proximal MCA segment as the reference on 5-mm MIP images. The advantage of the 5-mm MIP images over the 1 mm-thin slice images includes visualization of the entire MCA course on a single image [18]. Although the analysis of thin-section images would be cumbersome, especially in emergency cases, our method using MIP images created at 5 mm thickness and 3 mm intervals has advantages and overcomes the problems of thin-section images. In MIP images, the right and left MCA can be easily compared, and the number of MIP slices was almost the same as the 5 mm averaged images, which can be accepted in an emergency setting. The MIP images with a gold standard of CT angiography have showed better sensitivity of hyperdense sign. However, they have evaluated only a subjective assessment [14, 19, 20]. MIP images may facilitate radiologists and clinical physicians to detect hyperdense MCA sign [12, 21]. In MIP image, there is no need to closely examine all the slice set. Thus, evaluation of CT using MIP image could be a quicker, more effective, and more widely applicable tool for patients with acute focal neurological deficits.

A comparative strength of our study is that both experimental groups included a standard dataset and the images used for the quantitative analysis. On MIP images, the optimal threshold of the ratio on the affected and unaffected sides was 1.152, and this result could be applied to artificial intelligence-based research in the future.

This study has several limitations. First, this was a retrospective study, which might have affected the interpretation of the results. Second, the sample size was small. Third, the early CT signs other than hyperdense MCA sign was observed in five cases, which might affect the interpretation. Finally, the target was only the MCA, and acute embolization of the anterior cerebral artery or posterior cerebral artery was excluded from this study. Therefore, the hyperdense artery sign of these arteries should be examined in future studies.

In conclusion, the sensitivity of the hyperdense MCA sign on 5 mm MIP images was significantly higher than that on conventional 5 mm averaged CT images. Diagnosis using MIP images could be useful for the early initiation of proper therapy for patients with acute focal neurological deficits.
